# Affinage: Genome-Scale Mechanistic Gene Annotation from the Published Literature

**Published:** 2026-07-02

**Authors:** Matteo Di Bernardo, Iain M. Cheeseman

**Affiliations:** 1 Whitehead Institute for Biomedical Research, Cambridge, MA, USA; 2 Department of Biology, Massachusetts Institute of Technology, Cambridge, MA, USA.

## Abstract

Understanding the mechanistic function of a gene is a critical starting point for biology. However, for much of the human proteome that knowledge is scattered across thousands of primary papers or remains poorly established, while the curated databases biologists rely on can lag years behind recent literature. Large language models can now read and synthesize that literature on demand, but doing so faithfully for many genes is an expensive, non-reproducible retrieval session that does not scale across users. Here, we present Affinage, an LLM pipeline that performs this retrieval and mechanistic reasoning once per gene — from the primary literature alone — and stores the result as a reusable, structured annotation. A biologist-designed reading pass extracts only direct experimental evidence, and a synthesis pass reasons over those findings alone. Applied across the genome, Affinage annotates 19,293 human protein-coding genes. This analysis provides mechanism for thousands of genes whose UniProt function is empty or a stub, beating the curated reference on 99.1% of head-to-head genes as scored by a cross-family LLM judge. Affinage also delineates the ~10% of the proteome that remains mechanistically uncharacterized and will serve as a continuously-updated, literature-grounded census of gene function. All records are released openly at https://affinage.wi.mit.edu. More broadly, Affinage serves as an example of how domain experts can encode their expertise into scalable LLM pipelines to improve the publicly available data that guides biological hypotheses and experimentation.

## 1. Introduction

A gene’s function underlies nearly every experiment or hypothesis a biologist designs, and yet that knowledge is unevenly recorded: well-studied genes carry rich mechanistic literature while much of the proteome remains thinly described or not at all. Curated reference databases often trail primary literature and cannot carry mechanism at the level reasoning requires. UniProt functional descriptions (The UniProt Consortium, 2023) are bound to manual curation cycles, and Gene Ontology (Ashburner et al., 2000; Aleksander et al., 2023) cannot express the substrate, structural, or partner-level detail mechanistic reasoning demands. As a growing ecosystem of computational tools is built on this curated text — gene-representation models that embed it as features (Chen & Zou, 2025; 2024) and autonomous bio-AI agents that call it as a tool (Huang et al., 2025; Mitchener et al., 2025) — a more continuously updated and faithful representation of gene function is greatly needed.

Large language models (LLMs) can now read and synthesize the primary literature on demand (Li et al., 2025). But naively asking an LLM what a gene does carries three limitations. First, without carefully designed extraction criteria an LLM overclaims, reporting phenotypic associations or hypotheses as established mechanism. This erases the distinction between reasoning over evidence and over noise that a domain expert would enforce. Second, generation is non-deterministic: the same gene, queried again or by another user, yields a different answer, so no annotation is stable or reproducible. Third, grounding each query against the literature or live databases — increasingly through per-call tool and MCP access — is expensive, and that cost compounds across every gene and every user.

Here, we present Affinage, which performs this retrieval and mechanistic reasoning once per gene — under prompts designed by cell biologists to encode what counts as direct experimental evidence — and stores each result as a structured, literature-grounded record any user can query at lookup cost. We release all 19,293 human protein-coding records as a reusable base layer: a biologist can read a gene’s mechanism, and a downstream model or tool ecosystem can ingest it as a richer, more up-to-date substitute for a UniProt-derived feature, without re-deriving either.

## 2. Affinage pipeline

**Figure 1.**
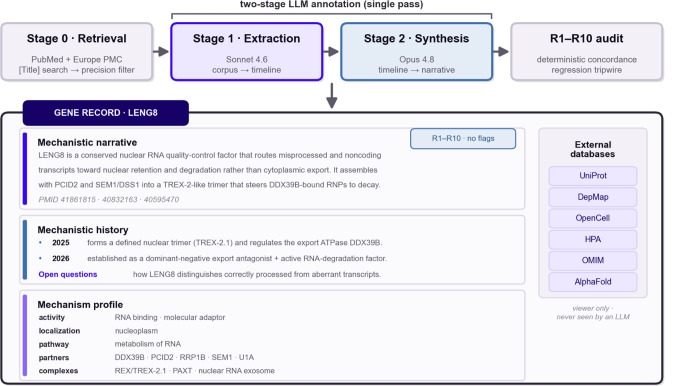
The Affinage pipeline and the record it produces. *Top:* Stage 0 retrieves and precision-filters a per-gene corpus with no LLM; Stage 1 (Sonnet 4.6) extracts indexed mechanistic findings; Stage 2 (Opus 4.8) synthesizes the narrative from the findings alone; a deterministic R1–R10 audit screens every record. *Bottom:* each gene yields one structured record — a declarative mechanistic narrative, the mechanistic history (the dated steps and the open questions outstanding at each one), and the mechanism profile.

Affinage is a single-pass, two-stage pipeline (Figure 1). For each gene, a deterministic retrieval stage (Stage 0, no LLM) first assembles a per-gene literature corpus and strips off-target papers. A reading pass (Stage 1) then extracts dated mechanistic findings, and finally a synthesis pass (Stage 2) reads those findings to produce the gene’s structured record. No curated-database content is ever shown to either LLM stage, and prefetched reference data is attached to each record for the viewer only. The deterministic audit layer (Section B) and the LLM-judge validity evaluation (Section 3) sit outside the generative chain. Full implementation, batch mechanics, and cost breakdowns are provided in Section A.

### 2.1. Stage 0: retrieval and precision filtering

A biologist exploring a gene’s function starts by searching the literature for its name. We reasoned that, for a holistic representation of a gene’s mechanism, a retrieval strategy would function more faithfully and inexpensively without an LLM in the loop. Therefore, Stage 0 is a focused retrieval strategy that captures the most relevant papers for a gene, and avoids pitfalls: that short symbols are ambiguous, that a gene’s older aliases still index real papers, and that a paper merely name-dropping a gene is not always about it.

Concretely, a title-restricted PubMed search (Sayers et al., 2022) over the canonical symbol and its HGNC aliases (Seal et al., 2023) assembles the corpus. A precision layer then drops the off-target papers that a search pulls in, including catalog papers that name many genes at once, and the literature of a different, unrelated gene whose symbol happens to collide with an alias. The corpus is then ranked such that the most gene-specific, well-supported evidence reads first (query construction, short-symbol disambiguation, and ranking are detailed in Section A). The title-first strategy is precision-weighted by design. However, a fallback abstract search (triggered when fewer than 10 papers are found for a given gene) recovers some of these. Even so, not every paper relevant to a gene is captured. A key paper that never names the gene in its title can be missed entirely, and because it never enters the corpus the miss is silent, placing it beyond the reach of the deterministic audit that only checks evidence provided to the LLM.

### 2.2. Stage 1: reading pass

The reading pass asks of each paper the question a biologist asks when triaging a stack of abstracts: does this paper actually establish something about how a gene works? Given the prevalence of noise and tendency to overclaim in biological literature, we designed the Stage 1 prompt to be strict: the ranked corpus is read by an LLM under a prompt that keeps a finding only when a direct experiment supports it. It admits substrates from co-immunoprecipitation or reconstitution, enzymatic activities from in-vitro assay or active-site mutagenesis, structures with functional validation, pathway positions from genetic epistasis, post-translational modifications mapped to residues, localization by imaging or fractionation — and excludes the rest: phenotypic associations, expression correlations, and unvalidated computational predictions. It also filters on epistemic status, so a claim a paper raises and refutes, a hypothesis floated in discussion, or a mechanism ruled out never enters the record. Each kept finding is stored with its experimental method, date, journal, and supporting PubMed IDs (PMIDs) (Section A). When the reading pass returns no findings, a recovery step distinguishes a genuine absence of mechanistic evidence from a symbol-retrieval failure: if the gene carries a distinctive UniProt protein name, Stage 0 re-retrieves by that name — surfacing literature indexed under the protein rather than the symbol — and the corpus is re-read.

### 2.3. Stage 2: synthesis pass

With the relevant findings selected, what remains is the synthesis a biologist would do by hand: assemble the vetted evidence into a coherent account of what the gene does. In our experience, decoupling the conclusions of individual papers from a synthesis that reads only those conclusions is critical to reasoning that faithfully reflects gene function. Stage 2 reads the evidence layer alone — never the abstracts — so it cannot reach for literature the reading pass did not admit. It returns the gene’s record in three layers: a declarative mechanistic narrative; a per-finding history that carries the open questions outstanding at the time of each study; and a structured mechanism profile placing the gene on controlled vocabularies for molecular activity, localization, pathway, named complexes, and named partners (Section D.1). The narrative is written under a fixed contract: declarative and unhedged, with uncertainty carried in the per-finding open questions rather than the prose, and length scaled to the available evidence so dense genes get more text and sparse ones are not padded (Figure S1).

## 3. Model selection and evaluation

With this structure in mind, we sought to answer two key questions: (1) How faithfully Affinage’s output traces back to the corpus it reads, and (2) how it improves over what a curated database like UniProt already offers. We addressed both questions — and chose the underlying models behind the pipeline — on a purpose-built, failure-enriched 100-gene cohort. This cohort includes ~50 of the hardest retrieval and grounding cases, of which 20 truly have no known mechanism, and ~50 clean controls stratified by corpus size (Section C.3). The refusal and flag rates for this cohort are deliberately pessimistic and not genome-representative — the cohort exists to stress the pipeline and keep configurations commensurable.

**Figure 2.**
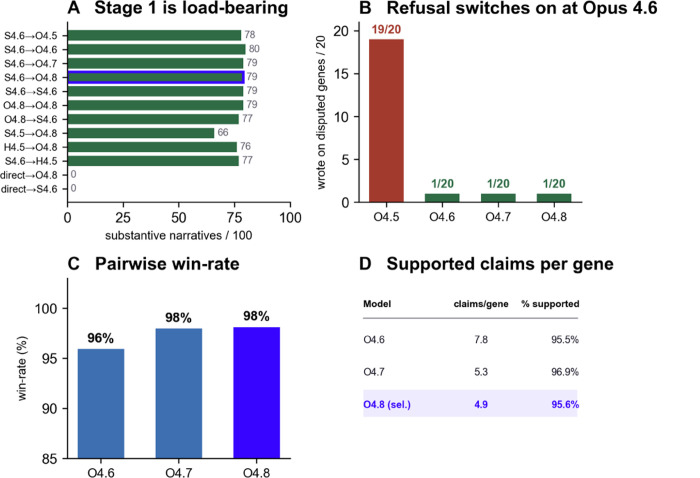
Model selection on the 100-gene cohort. (A) Stage 1 is load-bearing: two-stage configurations produce 77–80 substantive narratives per 100 genes, while the direct single-stage variants collapse to 0. (B) Refusal switches on at Opus 4.6: on the 20 disputed genes a model should decline, Opus 4.5 writes on 19/20 whereas Opus 4.6/4.7/4.8 each write on only 1/20. (C) Among the refusal-qualifying models, pairwise win-rate vs. UniProt is flat, so quality offers no basis to choose among them. (D) Supported claims per gene: the selected Opus 4.8 is the most consolidated (4.9 vs. 7.8 for Opus 4.6). Faithfulness (0.26% adjudicated error, 0 contradictions) and the memorization probes are reported in the text.

After tuning the retrieval Stage 0 over this challenging cohort, we swept the Stage 1 and Stage 2 generator assignments on this identical cached corpus, isolating the models from retrieval (Section C.4). We found that Stage 1 is load-bearing (Figure 2A): feeding the raw corpus straight to synthesis collapsed output to essentially zero (0/100) substantive narratives, against 77–80 for the two-stage configurations. The models also differed in restraint — whether they declined to write a narrative when the evidence did not support one, instead of fabricating a plausible mechanism. On the 20 cohort genes whose retrieved literature establishes no real mechanism (the correct output is no narrative at all), the older Opus 4.5 wrote a narrative anyway for 19/20, whereas newer models (Opus 4.6, 4.7, and 4.8) correctly abstained on all but one (Figure 2B).

To ground the trust and improvement over UniProt in a non-Anthropic model, we turned to a cross-family judge (Prometheus-8×7b, a Mistral-family model) that scores pairwise quality against the UniProt function field (blind, position-swapped) and per-claim faithfulness against the retrieved corpus (protocols in Sections C.1 and C.2). On quality, the three qualifying models (Opus 4.6/4.7/4.8) tied. Importantly, the win-rate over UniProt was unanimously high (Figure 2C), and per-claim faithfulness was likewise uniformly high across them (95–97% supported; Table 2). Adjudicating every flagged claim on the selected combination against its cited abstracts left 0.26% of the 388 cohort claims as genuine errors, with 0 contradictions. Quality could not break the tie, so we selected the latest combination: Sonnet 4.6 (reading) → Opus 4.8 (synthesis), the most consolidated of the qualifying combinations (4.9 vs. 7.8 supported claims per gene), which beats the UniProt function field 52/7/1 (wins / ties / losses) on the 60 swap-stable genes (Figure 2D).

A final pair of probes checked that the narratives came from the supplied corpus, not from the model’s memorized training data. In the empty-corpus probe, given no corpus at all, the model must trip the refusal sentinel, which it did successfully on 18/18 genes. In the wrong-corpus probe, given gene *A*’s identity header but gene *B*’s abstracts, a memorizing model would recite *A* and cite papers absent from the supplied corpus; across 18 probes none of the 18 narratives cited an out-of-corpus paper, with 10 grounded in the supplied (*B*) corpus and 8 refused the mismatch. With selection settled and grounding confirmed, we applied this configuration across the genome.

## 4. Genome-wide results

### Genome-scale resource.

Run across the genome, the winning Sonnet 4.6 → Opus 4.8 configuration yielded a mechanistic description for nearly every human protein-coding gene — including biology too recent or too sparse for curated databases to carry. The released database covers 19,293 of 19,296 HGNC protein-coding genes (99.98%): it contains 270,143 extracted mechanism findings (median 11 per gene), open mechanistic questions attached to each history step, 17,399 structured mechanism profiles (90.2% of annotated genes; Figure S2), and 71,112 literature-derived partner edges across 14,258 genes. The extracted mechanism literature skews recent and grows year on year — 33% of findings cite work from 2020 or later (Figure S3). The resource is served as a live REST API and MCP endpoint at https://affinage.wi.mit.edu (Section D.2).

### Genome-scale validation.

**Figure 3.**
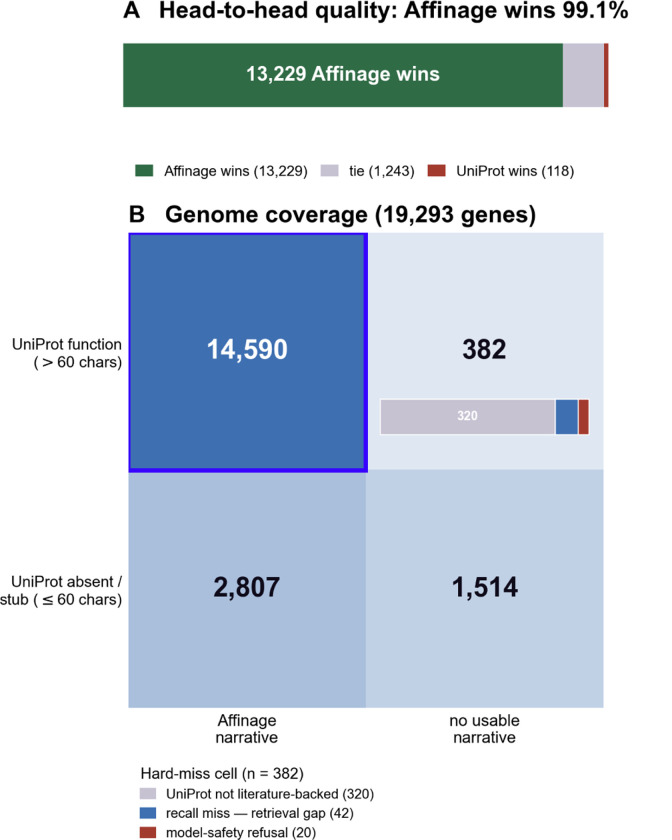
Affinage versus UniProt. (A) Head-to-head pairwise quality (cross-family judge, blind, position-swapped) on the 14,590 genes where both carry substantive content: Affinage wins 99.1% of decided pairs. (B) Coverage cross-tab of all 19,293 genes by UniProt function (> 60 chars) vs. usable Affinage narrative; the violet outline marks the head-to-head set in (A). The 382 hard misses are further partitioned into 20 model-safety refusals, 42 recall misses, and 320 correct non-writes where UniProt’s own function is not literature-backed.

To both evaluate and ground the genome-wide run, we extended the Prometheus pairwise and faithfulness evaluations introduced on the cohort (Section 3) to every released record. Overall, we found that results in our test cohort extended to show Affinage consistently matching or beating UniProt. On the 14,590 genes where UniProt function text > 60 characters, the cross-family judge favored Affinage 13,229 / 1,243 / 118 (wins / ties / losses) — a 99.1% win rate over decided pairs (Figure 3A). The narratives also stay grounded: run over the entire resource, the per-claim faithfulness screen scored 97,111 claims across 17,360 genes at 97.95% supported and 0.029% contradicted (28 claims), an unadjudicated 2.05% flag rate that upper-bounds the error. Beyond the LLM judge, a large majority of records also cleared a deterministic structural audit (R1–R10; pure regex and SQL, no LLM): only 206 of 19,293 records (1.07%) tripped at least one flag, most often a cited PMID absent from the shown corpus, with the per-rule breakdown in Section B.

The genome-wide coverage cross-tab (Figure 3B) splits all 19,293 genes by whether UniProt carries a substantive function and whether Affinage writes a usable narrative: 14,590 carry both (the head-to-head set scored in A), Affinage adds a narrative for 2,807 genes whose UniProt function is empty or a sub-60-character stub (Figure S1), and 1,514 have neither. The 382 genes with a UniProt function but no Affinage narrative are the apparent misses. We further partitioned these into 20 model-safety refusals, 320 correct non-writes where UniProt’s own function is homology-inferred, unattributed, or curator-only — not itself literature-backed — leaving 42 true recall misses (R5), where UniProt’s function is experimentally backed and on-target literature sat in the retrieved corpus yet nothing was extracted. Across the genome, Affinage matches or beats the curated reference wherever both exist and extends mechanism well beyond it.

## 5. Limitations: abstract-only vs. full-text reading

The single largest limitation is that Affinage reads abstracts, not full text: mechanistic detail living only in a paper’s methods, results, or supplement is invisible to the reading pass, and synthesis cannot produce what reading did not extract. The limitation is bounded, not open-ended — the faithfulness eval scores any claim overreaching the corpus as unsupported, so abstract-only reading caps recall but does not silently inflate precision. However, unlike more complex biological AI tools that seek to dive deep into a specific biological question, where specific methods, results, and figures may be critical, we postulated that for a more high-level task, digesting just abstracts would capture the majority of the mechanism, and that the recall gap would not translate into quality. To test this, we ran a full-manuscript reading pass on the 100-gene cohort, with a 1M-token context window to capture as much of the full text as possible. We found that this task, in itself, was challenging due to known paper paywalls — we only retrieved ~46% of cited papers via PMC, Europe PMC, and Unpaywall. Full text did lift recall — +16.2% more extracted findings (1,075 vs. 925 on the 76 genes substantively annotated under both corpora) — but at ~15× the Stage-1 token cost, and the gain did not translate into quality: a cross-family pairwise judge rated the full-text and abstract-only narratives a statistical tie (20 wins / 33 ties / 24 losses vs. the abstract-only approach). For genome-scale mechanistic annotation, abstract-only reading is therefore the right default. A retrieval path that targets the specific results sections carrying novel mechanism is the more promising future direction.

## 6. The uncharacterized genome

Affinage contributes a genome-scale, literature-grounded mechanistic annotation of the human proteome: one structured record per gene, synthesized only from primary literature, validated against both the corpus and the UniProt reference, and released openly. It is most useful on genes that curated databases miss — those whose mechanism is too recent or too scattered across the literature to have entered a curated function field. Below, we provide several example case studies.

LENG8 is one such gene. UniProt lists it as “Leukocyte receptor cluster member 8” with no function entry, and yet all five of its mechanistic papers appeared in 2025–2026. Affinage resolves LENG8 as a conserved nuclear RNA quality-control factor that, assembled with PCID2 and SEM1 into the REX complex, acts as a dominant-negative antagonist of TREX-2, diverting polyadenylated transcripts from nuclear export toward exosomal degradation. The Affinage record also carries the questions those papers resolved and the ones still open (e.g. the structural basis for recognizing misprocessed transcripts).

Across unrelated biology, Affinage produces equally detailed accounts of gene function. It resolves KHNYN as a Mn^2+^-dependent endoribonuclease effector of the ZAP antiviral system that cleaves CpG-enriched viral RNA to restrict HIV-1, SARS-CoV-2, and influenza, and reconstructs EEPD1’s dual role as a structure-specific nuclease channeling stalled replication forks into homologous recombination and a myristoylated membrane enzyme activating PKA, tying its loss to cGAS–STING signaling. It identifies CDK19 as a Mediator-module kinase that also acts kinase-independently in interferon responses, tracing its *de novo* variants to a syndromic neurodevelopmental disorder with epileptic features, and assembles ANKRD22’s coupling of mitochondrial metabolism to innate immune signaling through MAVS and NIK. Spanning antiviral defense, replication-fork repair, transcriptional control, and metabolic–immune coupling, none of these genes carries a UniProt function field — each is a new mechanistic description Affinage built entirely from literature that postdates the curated record.

Importantly, Affinage finds that 1,896 human protein-coding genes (9.8%) still yield no usable mechanistic narrative. For most genes, this is because the published record does not yet establish how they work. This residual is itself a result: a continuously-updated, literature-grounded census of the protein-coding genes that remain mechanistically uncharacterized, re-derived as new papers appear. Nearly a tenth of the human genome is still functionally uncharacterized, and that boundary is now trackable gene by gene as the literature advances.

## 7. Conclusions

Affinage shows that a single-pass, two-stage LLM pipeline — read the abstract corpus, then synthesize — produces genome-scale mechanistic annotation that is both literature-grounded and validated, not merely fluent. The released resource is one instance of a broader recipe: a subject-matter expert encodes what counts as evidence into an LLM pipeline, runs it once at scale, and releases the result openly, turning a one-off query into a reusable, citation-anchored dataset the field can build on. As LLM-assisted reasoning becomes routine, this strategy of domain experts using LLMs to produce high-quality, openly-available data may be among the most powerful ways to raise the quality of the data the field reasons over.

## Supplementary Material

Supplement 1

## Data Availability

All gene records, a REST API, and an MCP server are available at https://affinage.wi.mit.edu.^1^ Source code: https://github.com/cheeseman-lab/affinage. The reading pass runs Claude Sonnet 4.6 and the synthesis pass runs Claude Opus 4.8; the genome was annotated May 2026 and is reproducible from the released code, which versions both the extraction and synthesis prompts. Beyond the source repository, the full extraction, synthesis, and Prometheus judging prompts are also rendered on the site’s About page for direct inspection. The evaluation environment, including the Prometheus-8×7b judge, is pinned for reproducibility (vLLM 0.10.2, PyTorch 2.8.0, Transformers 4.55.2), and the validation cohort is seeded.

## A. Pipeline and retrieval mechanics

Genome-scale execution chunks the HGNC protein-coding universe (Seal et al., 2023) into batches of ≤ 2,000 genes, sized by the batch API’s payload limit (~256 MB) against the observed mean of ~80 KB of formatted prompt per gene; larger chunks are rejected at the edge. Each chunk runs sequentially through retrieval, the reading pass, and the synthesis pass, then upserts into the database. Chunks are independent: a failed chunk does not block the next, and because the upserts are idempotent an interrupted run can simply be relaunched.

Retrieval is the wall-time long pole. Each gene’s corpus and reference metadata are disk-cached, and a gene whose cache already exists is skipped, so partial reruns reuse prior PubMed (Sayers et al., 2022) and iCite (Hutchins et al., 2016) work rather than re-querying the APIs. Fresh retrieval runs at ~50 minutes per 2,000-gene chunk and a warm cache reduces this to ~5 minutes, making interrupted-run restarts effectively free. All NCBI requests share a single rate limiter that holds the published request budget across the parallel retrieval and prefetch workers, and per-gene failures (transient NCBI errors, schema-invalid synthesis output) are logged and skipped so the run continues.

The title query searches the canonical symbol plus up to five HGNC aliases (covering both previous and current gene symbols) and is filtered by biological terms to suppress non-biomedical hits; short symbols (≤4 characters) receive additional disambiguated queries (“{gene} protein” and the UniProt full name), since short names collide with common abbreviations, and if the title search returns fewer than ten papers it broadens to title-or-abstract, recovering recall for genes too sparsely studied to be named in a title. bioRxiv/medRxiv preprints are added via Europe PMC (Europe PMC Consortium, 2015). The precision layer’s catalog denylist is seeded with 262 PMIDs that each appear in more than 50 genes’ corpora (8.2% of corpus slots) and is recomputed per run to track new catalog papers; for a gene whose alias is itself another gene’s canonical symbol, the query is restricted to the canonical plus previous symbols, stopping the collision at the fetch step. Survivors are ranked by inverse gene fan-out, then by whether the gene is the title’s primary subject, with peer-review status and iCite citation count as tiebreaks; HGNC serves here only as a naming authority, never as a curation set.

Each finding the reading pass keeps is recorded as a structured evidence entry with a two-axis confidence score — method tier × evidence preponderance — so the synthesis pass can weight stronger evidence over weaker.

### Index-based citation.

The synthesis pass cites each claim by the integer index of the finding it rests on, never by writing out a reference identifier; a deterministic post-processing step then resolves each index to the PMIDs recorded for that finding. Because the model never emits a citation token along this path, a confabulated or truncated reference cannot arise from index resolution — removing the dominant failure mode of literature-grounded generation for every citation routed through the index. The residual, a raw PMID emitted outside the index path, is what the audit layer monitors (Section B).

### Incremental maintenance.

The pipeline supports an incremental-rerun mode for keeping the resource current as new literature appears. On a rerun, retrieval re-fetches each gene’s corpus rather than serving it from cache, then compares the new reference set against the set stored on the prior record: genes with no new papers are copied forward at zero model cost, and only genes with at least one new paper re-enter the reading and synthesis passes. Idempotent upserts let an interrupted maintenance pass be relaunched without duplicating work.

### Cost.

Costs are reported at the batch rate (50% of on-demand; Anthropic 2025). Synthesis dominates spend: the Opus synthesis pass costs ~2.4× the per-gene reading pass, with the per-gene total amortizing to a mean of $0.13 (median $0.11) and a total genome-scale spend of $2,505. The deterministic audit layer (Section B) calls no API and runs in ~30 seconds over the read-only database.

## B. Deterministic audit layer

The audit layer is a deterministic regression tripwire, not a measure of semantic correctness: it catches structural failures that should not survive the single-pass pipeline. Every rule is pure regex and SQL with no LLM in the evidentiary chain, reading only the narrative text, the HGNC alias table, the extracted finding timeline, and the length of the prefetched UniProt function field — never any curated-database content, preserving the database-exclusion invariant. The ten rules fall in three tiers; Table 1 gives what each catches and how often it fires across the released resource, and the full per-rule specifications are published alongside the resource (https://affinage.wi.mit.edu).

The IDENTITY tier (R1–R4) catches a narrative about the wrong gene, or the wrong product of the right gene: an alias that is itself another gene’s canonical symbol dominating the text, an opening led by a non-human organism homonym, a first noun phrase naming a different symbol, or an opening dominated by a non-coding product of the locus.

The GROUNDING tier (R5–R8) catches a narrative that under-extracts or misuses evidence: a refusal placeholder despite substantive UniProt function text, citations that barely overlap the gene’s independently indexed literature (a corroborating signal only), a cited reference absent from the shown corpus (a truncation or fabrication), or too large a fraction of uncited body claims.

The BEHAVIOR tier (R9–R10) catches model-behavior anomalies: a failed contamination probe, or no usable narrative at all — a model-safety refusal, a parse failure, or an over-refusal. A placeholder with zero findings is the correct outcome for an empty timeline and is never flagged.

R7 is the most frequently tripped rule (66 records, 0.34%): a PMID cited in the narrative but absent from the shown corpus. In these cases the underlying claim is grounded in a real corpus paper, but the rendered identifier is mis-attributed or truncated — a citation-grounding failure, not a wrong-gene error. The next is R5 (42), the deterministic recall miss — a gene with experimentally-backed UniProt function and on-target corpus evidence that yielded no narrative; R8 flags a further 28 uncited-synthesis cases. The IDENTITY tier (R1–R4) flags 46 records and BEHAVIOR (R9–R10) 27, the latter (R10, 0.14%) dominated by model-safety refusals enriched for host–pathogen and viral-entry genes.

**Table 1.** Deterministic structural audit (R1–R10). What each rule catches and its flag count across the released genome-scale resource; reproducible from the database alone, no LLM. Counts are per rule, so a gene tripping several rules is counted under each; R6 only corroborates an IDENTITY call and never escalates alone.

Tier	Rule	Fires when	Genes
IDENTITY	R1	alias dominates the narrative *and* the contamination reaches it	26
	R2	opening led by a non-human organism qualifier	0
	R3	opener names or equates the gene to a different HGNC symbol	11
	R4	opening dominated by a non-coding product of a protein-coding locus	9

GROUNDING	R5	no narrative despite experimental UniProt + on-target corpus	42
	R6	citations barely overlap the gene’s indexed literature	8
	R7	cited PMID absent from corpus (truncation / fabrication)	66
	R8	uncited body-claim fraction > 0.30	28

BEHAVIOR	R9	contamination probe failed (empty- or wrong-corpus)	0
	R10	no usable narrative (refusal / parse-failure / over-refusal)	27

**Records flagged (any rule)**			206 (1.07%)

## C. Faithfulness and pairwise evaluation

Both the faithfulness and pairwise judgements use a deliberately cross-family LLM judge (Prometheus-8×7b, Mistral family) so there is no self-preference bias toward the Anthropic generators; the judge never sees any curated database, matching the pipeline’s own database-exclusion invariant. The full judging rubrics, like the extraction and synthesis prompts, are published alongside the resource (https://affinage.wi.mit.edu).

### C.1. Faithfulness rubric

In the absolute (faithfulness) mode the judge reads each decomposed narrative claim with the gene’s retrieved corpus and scores it on a three-level rubric (supported / unsupported / contradicted); open-question claims are routed out deterministically before scoring. An automated judge over-flags this task — it effectively scores a multi-cited claim against only the first of its cited abstracts and treats a paper indexed under a gene synonym as a “different gene” — so we use it as a high-recall screen and manually adjudicate every flagged claim against the full set of its cited abstracts. On the cohort almost all flags resolve to supported once that full set is read (e.g. IMP4/Mpp10, ZKSCAN1, STAG1, GTF3C2, TTC7B); the rare genuine error is a paralog or gene-family mis-attribution introduced at retrieval (e.g. SLC16A4 carrying SLC16A3/MCT4 glycolytic biology, its corpus legitimately co-mentioning the SLC16 family), and contradictions are absent. The screen’s run-to-run movement is itself within per-claim judge noise, whereas the adjudicated error rate is stable across resamples — which is why the adjudicated rate, not the raw screen, is the headline. At genome scale the same screen runs unadjudicated; the contradiction rate, which needs no adjudication, is the load-bearing signal there.

### C.2. Pairwise protocol

The relative (pairwise) mode presents the judge with two descriptions of the same gene and asks which is better supported by the retrieved corpus — here the Affinage narrative against the UniProt function field, on genes carrying both. Each pair is source-blind (the judge is not told which side is Affinage) and scored under both presentation orders (position swap) to control for order bias; a verdict counts only when it is stable across the swap. The win/tie/loss outcome is reported in the body and Figure 2B.

### C.3. Validation cohort

Model selection and validation use a purpose-built, failure-enriched 100-gene cohort (seeded for reproducibility). Failure-class genes are drawn by audit-subtype quota (alias collision, alternate product, paralog, recall miss, uncited synthesis) plus over-refusal probes; the clean controls are stratified by corpus size (sparse < 5, moderate 5–20, rich > 20 papers). The identical set runs through every configuration and comparison. Re-classified against the current audit rules, the realized composition is 57 failure-class and 43 control genes (a few build-time controls trip a current rule).

### C.4. Model and architecture ablation

The full per-variant sweep summarized in Figure 2 crosses the reading- and synthesis-stage generator assignments on the identical cohort and cached corpus, holding the database-exclusion invariant fixed; the deterministic audit-layer counts (substantive narratives, refusals, structural flags) are the discriminating selection axis. Table 2 reports the cross-family judge on the same configurations. The configurations cluster tightly — supported rates and pairwise wins over UniProt span only a few points, within the per-claim judge noise — so the judge corroborates rather than drives the choice. Selection rests on the deterministic axes (refusal discipline and contract adherence), where the winning combination is clearly strongest and also carries the joint-lowest adjudicated error rate.

**Table 2.** Cross-family judge across configurations (100-gene cohort). The supported-rate column is the per-claim screen before manual adjudication (see Section C.1); the vs. UP column is pairwise win/tie/loss against UniProt over swap-stable genes. Differences are within judge noise; the configuration is selected on the deterministic axes.

Stage 1 → Stage 2	Supp.%	vs. UP
S4.6 → O4.8 (winning)	95.6	52/7/1
S4.6 → O4.5	94.8	51/11/6
S4.6 → O4.6	95.5	47/12/2
S4.6 → O4.7	96.9	48/11/1
S4.6 → S4.6	96.8	52/7/1
S4.6 → H4.5	96.0	53/5/1
S4.5 → O4.8	97.7	44/6/1
H4.5 → O4.8	96.7	52/6/1
O4.8 → O4.8	98.1	54/5/2
O4.8 → S4.6	97.8	48/11/1

## D. The resource

### D.1. Output layers

Each gene record exposes three synthesis layers, all produced by the synthesis pass from the extracted findings alone. The mechanistic narrative is the declarative synthesis described in the body — the closest analog to a UniProt function field, but with per-finding citation provenance and recent biology integrated. Its length is calibrated to the empirical distribution of UniProt function-field lengths rather than a fixed cap, so output scales with the depth of available evidence. The mechanistic history is a per-finding record of what each finding established, together with an explicit gaps field carrying the open questions outstanding at the time of each finding — the sole home for uncertainty, which is banned from the narrative. The mechanism profile places each gene on controlled vocabularies across the molecular-activity, localization, and pathway axes.

### D.2. API and MCP access

The resource is served as a live public REST API and an MCP server at https://affinage.wi.mit.edu, exposing per-gene records (all three synthesis layers plus the attached reference data) for programmatic and agent access. The deterministic audit flags are surfaced per gene through a dedicated endpoint and MCP tool, so a consumer can read the structural-QC status alongside the annotation. Symbol lookups are alias-aware: a query against a previous or non-canonical symbol resolves to the canonical record via HGNC (for example, ZNRD1 redirects to POLR1H), so callers reach the right gene regardless of which historical symbol they hold.
