# Supplementary material for: Affinage: Genome-Scale Mechanistic Gene Annotation from the Published Literature
Source: ArXiv. 2026 Jul 2:arXiv:2607.02217v1. Preprint. [Version 1] (PMC13345584)
Supplement: Supplement 1 [file NIHPP2607.02217v1-supplement-1.pdf]

E. Supplementary figures

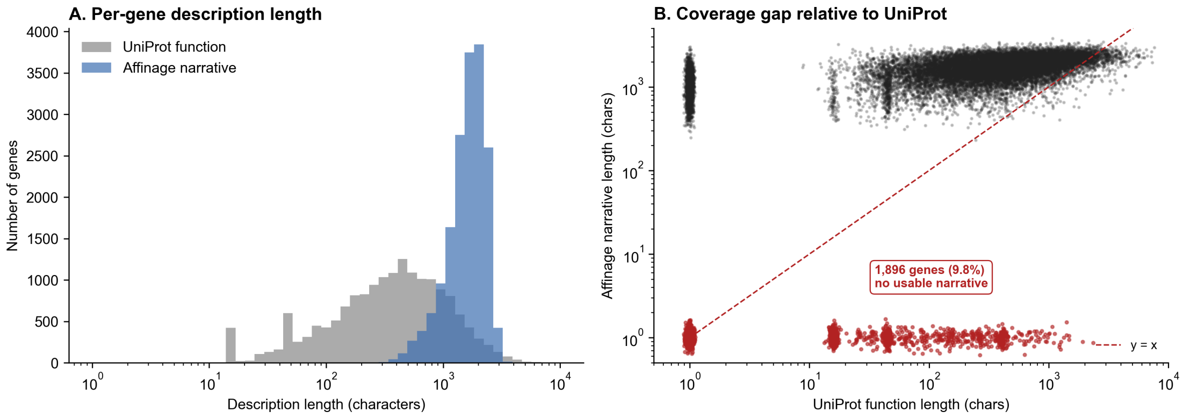

Figure S1. **Per-gene description length, Affinage versus UniProt.** (A) Length distributions over genes with usable content. (B) Per-gene scatter; the 1,896 genes with no usable narrative (9.8%) are floored to $y = 1$ (red).

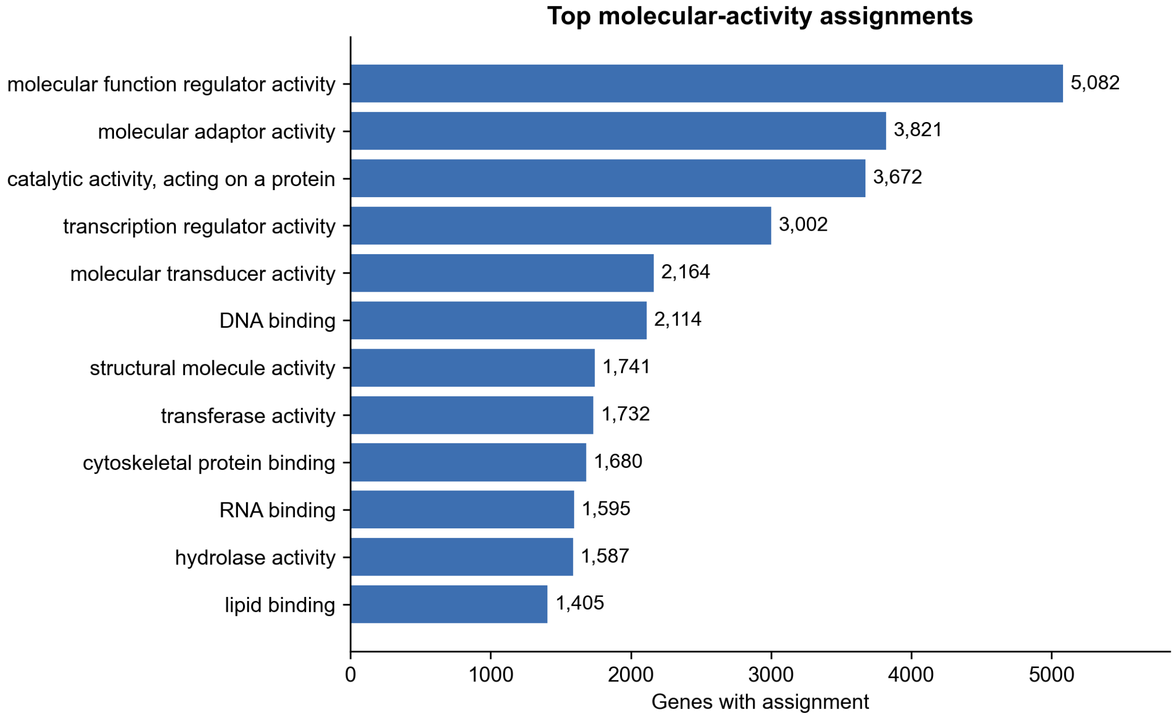

Figure S2. **Most frequent molecular-activity terms.** Top controlled-vocabulary molecular-activity assignments across the resource; “molecular function regulator activity” leads at 5,082 genes.

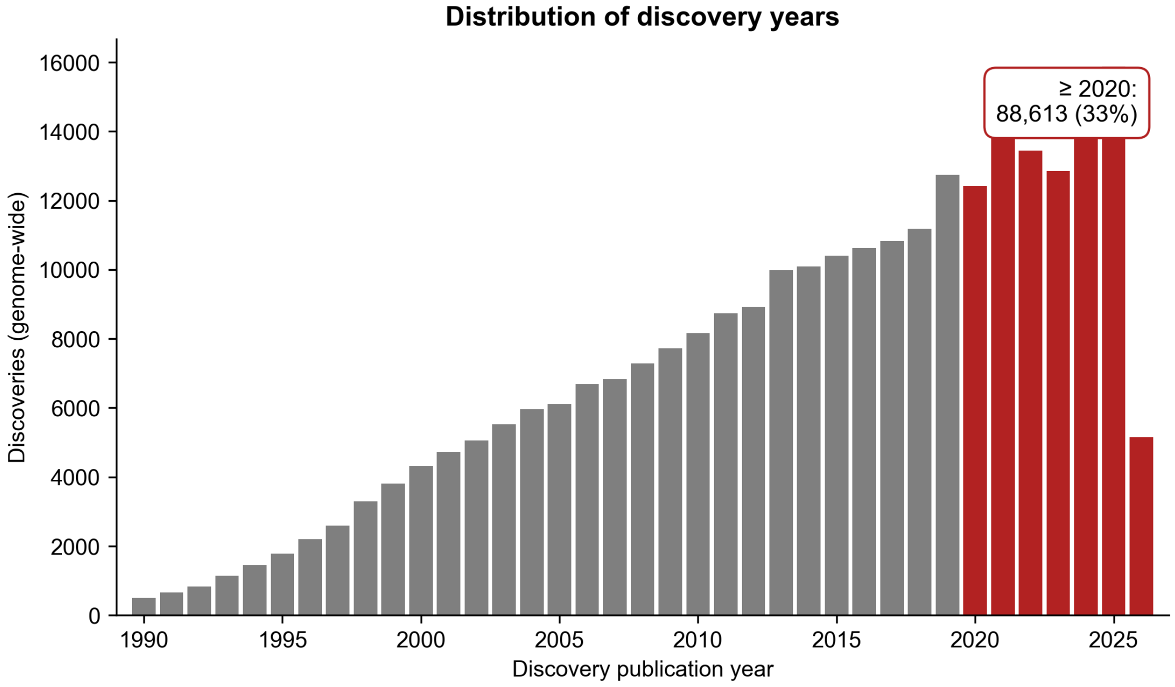

Figure S3. **Recency of the extracted mechanism literature.** Publication-year distribution across the 270,143 extracted findings; 32.8% (88,613) cite literature from 2020 or later.
